# CO_2_ Recycling to Dimethyl Ether: State-of-the-Art and Perspectives

**DOI:** 10.3390/molecules23010031

**Published:** 2017-12-24

**Authors:** Enrico Catizzone, Giuseppe Bonura, Massimo Migliori, Francesco Frusteri, Girolamo Giordano

**Affiliations:** 1 Department of Environmental and Chemical Engineering, University of Calabria, Via P. Bucci, 87036 Rende (CS), Italy; massimo.migliori@unical.it (M.M.); ggiordaunical@yahoo.it (G.G.); 2 CNR-ITAE “Nicola Giordano”, Via S. Lucia Sopra Contesse 5, 98126 Messina, Italy; giuseppe.bonura@itae.cnr.it (G.B.); francesco.frusteri@itae.cnr.it (F.F.)

**Keywords:** CO_2_ hydrogenation, dimethyl ether, low-carbon processes, thermodynamics, catalysis, zeolites

## Abstract

This review reports recent achievements in dimethyl ether (DME) synthesis via CO_2_ hydrogenation. This gas-phase process could be considered as a promising alternative for carbon dioxide recycling toward a (bio)fuel as DME. In this view, the production of DME from catalytic hydrogenation of CO_2_ appears as a technology able to face also the ever-increasing demand for alternative, environmentally-friendly fuels and energy carriers. Basic considerations on thermodynamic aspects controlling DME production from CO_2_ are presented along with a survey of the most innovative catalytic systems developed in this field. During the last years, special attention has been paid to the role of zeolite-based catalysts, either in the methanol-to-DME dehydration step or in the one-pot CO_2_-to-DME hydrogenation. Overall, the productivity of DME was shown to be dependent on several catalyst features, related not only to the metal-oxide phase—responsible for CO_2_ activation/hydrogenation—but also to specific properties of the zeolites (i.e., topology, porosity, specific surface area, acidity, interaction with active metals, distributions of metal particles, …) influencing activity and stability of hybridized bifunctional heterogeneous catalysts. All these aspects are discussed in details, summarizing recent achievements in this research field.

## 1. Introduction: How Can CO_2_ Become the Future Carbon Source?

Carbon dioxide is recognized as the main responsible of the super green-house effect, causing global warming and climate change. In this concern, to avoid more dangerous consequences, the Intergovernmental Panel on Climate Change (IPCC) and the United Nations Climate Change Conference (COP21, Paris, 2015) have emphasized the need to reduce CO_2_ emissions by at least one half of the current value by 2050, aiming at limiting the global average temperature increase to a maximum of 2 °C [1]. Carbon dioxide is mainly emitted from power plants (e.g., coal-based) and vehicles, but also other industrial sources contribute to increase the CO_2_ emission into the atmosphere, such as boilers or cement and steel plants [2]. The growing world population, the enhancement of welfare, the change in food habits are also causing an increase of animal farms so boosting CO_2_ emissions [3]. Aiming at reducing CO_2_ emissions, a carbon tax has been proposed from several countries as a strategy able to balance the incremental costs of reducing carbon emissions with the incremental benefits for limiting damages due to climate changes causing additional costs for industry [4].

During the last decades, several strategies and technologies have been developed concerning capture and storage of carbon dioxide (CCS) and, by 2020, the number of projects dealing with this topic is expected to double even if few large-scale CCS plants are already working [2]. On the other hand, during the last years, the scientific community started to consider CO_2_ not as an expensive waste (especially in the countries where carbon taxes are applied) but mainly as potential carbon source, alternative to the fossil-ones. Therefore, the future perspectives of carbon dioxide emissions reduction will not only concern the development of more efficient CCS technologies but will involve new strategies development for CO_2_ recycling to energy vectors and chemical intermediates. In this concern, the conversion of CO_2_ to dimethyl ether (DME) has received renewed attention since DME can be used as an intermediate to produce several value-added products (gasoline, aromatics and olefin) or as an alternative fuel as detailed below [5]. 

DME, the simplest of the ethers, is a neither toxic nor carcinogenic molecule with a boiling point of −25 °C, but it is a liquid at room temperature under a relatively low pressure (≈0.5 MPa) [6]. Chemical and physical properties of DME are close to liquid petroleum gas (LPG) and published studies suggested that the technologies developed for storage and transport of LPG can be easily converted to accommodate DME with similar safety guidelines and codes [7]. DME is also an important chemical intermediate for production of widely used chemicals, such as diethyl sulphate, methyl acetate and, as mentioned before, light olefins and gasoline [8]. Nowadays, DME is mainly used as aerosol propellant in spray cans, replacing the banned ozone-destroying chlorofluorocarbon (CFC) compounds but, in the last decades, it is receiving a growing attention also as alternative and eco-friendly fuel. In 1995, an extensive collaborative research effort by Amoco (currently BP), Haldor Topsøe and Navistar International Corporation, demonstrated that DME could be a reliable alternative fuel for diesel engines with low-emission of NOx, SOx and particulate, to be produced at large-scale from methanol through a dehydration step [9]. These studies renewed attention on the outstanding performances of DME as alternative fuel to diesel and showed total compliance with the hardly strict California ultra-low emission vehicle (ULEV) regulations for medium-duty vehicles. Because of the large scale changes in fuel infrastructure, the global implementation of DME to power vehicles still remains an open challenge. Indeed, the primary DME market was the blending of DME with LPG and Amoco patented a DME/LPG blend for automotive applications [10], while the other future market perspectives of use of DME as fuel are: (1) alternative fuel for diesel engines; (2) fuel for power generation in gas turbine plants; (3) chemical intermediate for olefins and synthetic gasoline production. Therefore, rather than methanol, dimethyl ether can be considered both as reliable energy vector of the future and as chemical intermediate in low-carbon processes. In this concern, carbon dioxide can be used as reactant to produce methanol and then DME. Specifically, methanol is first produced from the hydrogenation of carbon dioxide, according to the following reaction:

CO_2_ + 3H_2_ = CH_3_OH + H_2_O

Then, DME is formed via alcohol dehydration:

2CH_3_OH = CH_3_OCH_3_ + H_2_O

By considering the reverse water shift reaction:

CO_2_ + H_2_ = CO + H_2_O

The global reaction leading to the formation of DME is:

2CO_2_ + 6H_2_ = CH_3_OCH_3_ + 3H_2_O

As stoichiometry suggests, six moles of hydrogen are necessary per mole of DME and there are no real advantages to adopt this pathway to produce DME (or even methanol) via CO_2_ hydrogenation, since hydrogen is usually produced from fossil hydrocarbons (mainly from natural gas or light hydrocarbons). Therefore, only if hydrogen is produced from non-fossil sources, the Methanol/DME Economy Theory will be a reliable option; in particular, if hydrogen is directly produced using renewable energy sources, carbon dioxide hydrogenation will become a valuable strategy for renewable energy utilization in both chemical industry and power generation. Hydrogen can be produced from renewables in several ways. The current approach is the production of electrical energy by using renewable energy sources (e.g., solar energy) and the use of this energy for water electrolysis using Fuel Cells [11]. Other approaches to hydrogen production were also investigated: hydrogen from cyanobacteria or algae [12,13], biomass thermo-chemical process or anaerobic fermentation [14,15] or water splitting via photo-electrolysis [16,17].

**Figure 1:**
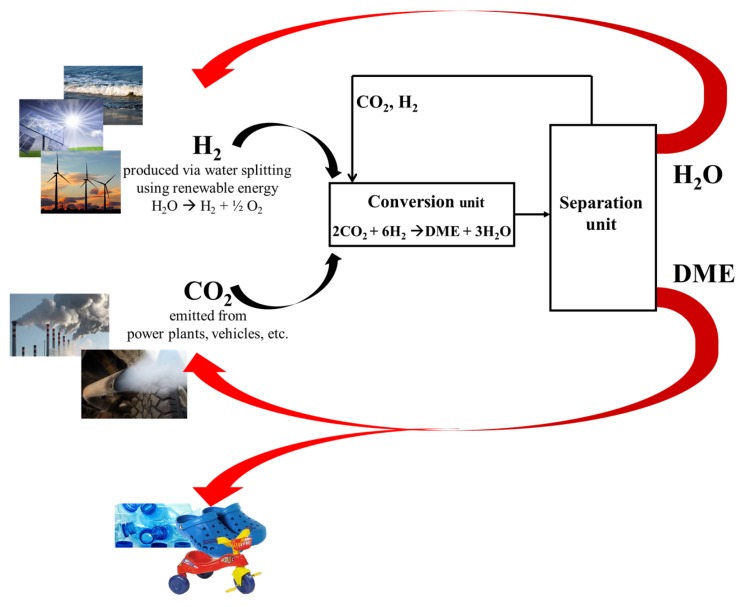
Proposed carbon cycle loop involving CO_2_ as energy vector.

Although hydrogen production from renewables remains an open challenge, the carbon-cycle based on CO_2_ Hydrogenation can be summarized in five steps (Figure 1):

(a) Production of hydrogen via water splitting by using renewable energy (e.g., solar energy);
(b) Capture and safe storage of CO_2_ from power plants emission or even from atmosphere;
(c) Hydrogenation of captured CO_2_ to produce methanol and/or DME (DME should be preferred because of its no-toxicity);
(d) Utilization of DME for energy production or as intermediate in the chemical-chain industry;
(e) Reuse the carbon dioxide from eco-friendly combustion of DME to re-produce itself.

Following this strategy and increasing the research on the listed steps, it will be possible to create an efficient CO_2_-based production system for both chemicals and energy production, compromising between energy request (also reducing the dependence on fossil sources) and socio-environmental safety (lowering the carbon dioxide emission into the atmosphere) [11]. Among the several challenges still open, despite several works have been carried out during the last years, the development of a highly efficient catalyst for CO_2_ hydrogenation is still a stimulating challenge. Since the formation of DME via one-pot CO_2_ hydrogenation involves two reaction steps (methanol formation and dehydration), the catalyst should exhibit a redox function able to hydrogenate CO_2_ to alcohol and an acid function able to convert the alcohol in the ether. Several strategies have been proposed in order to create a catalyst able to produce DME via one-pot hydrogenation of CO_2_ with good performances in terms of CO_2_ conversion, DME selectivity and stability. Recently, Álvarez et al. [18] discussed some catalytic aspects concerning one-pot CO_2_-to-DME process, revealing that further advances in research are necessary to reach high catalytic activity. In fact, despite Cu-based catalyst is expected to remain the most efficient catalyst for CO_2_-to-methanol reaction step, some aspects about bifunctional catalyst such as: (i) the choice of the acid function, (ii) the method used to prepare hybrid catalyst, (iii) the copper particle sintering and (iv) the catalyst deactivation, remain the main open issues in view of an optimization of the process. 

After a brief discussion about the use of DME as alternative fuel and thermodynamics of CO_2_-to-DME process, this paper focus on the critical evaluation of proposed catalytic systems, paying particular attention to the effects of both physicochemical properties on redox/acid functions and preparation method on catalytic performance of hybrid catalyst, emphasizing on the potential of zeolites as efficient acid catalysts for methanol dehydration step.

## 2. DME as Valuable Fuel of the Future

Since the middle of 1990s DME has been identified as a reliable diesel alternative for auto transportation. A chemical-physical property comparison between diesel fuel and DME is reported in Table 1, allowing one to identify both the advantages and disadvantages of using DME as an alternative fuel in such engines. A lower boiling point leads to a faster evaporation when liquid DME is injected into the engine cylinder, improving the combustion. In addition, a lower auto-ignition temperature allows one to obtain a higher cetane number for DME than that exhibited by diesel fuel. Generally, a high cetane number results in easy ignition, more complete combustion and cleaner exhaust gases; in addition, a high cetane number allows one to reduce not only noise but also fuel consumption and exhaust gas emissions during engine warm-up [6,19]. In addition, based on the similar chemical-physical properties between DME and LPG, existing infrastructure as the storage vessels and fuel lines used in LPG-based systems can also be used for DME. 

On the other hand, some disadvantages should also be taken into account to complete the evaluation of DME as substitute of diesel: DME exhibits a much lower LHV than diesel fuel (27.6 MJ/kg vs. 42.5 MJ/kg) and, for this reason, a larger amount of injected volume and longer injection period for DME is necessary in order to deliver the same amount of energy. Other disadvantages are related to the necessity to change the engine configuration if diesel fuel is substituted with DME. In fact, the lower viscosity of DME requires special gaskets to prevent fuel leakage. In addition, DME can act as a solvent for some organic compounds causing incompatibility with elastomers and plastic materials. Therefore, a careful selection of materials is required when DME is used as fuel in diesel engines [20].

The diesel-fueled compression ignition (CI) engine offers several advantages compared to a gasoline-fueled spark ignition (SI) engine (e.g., better fuel economy, higher power performance, and longer expected life).

Nevertheless, CI engines have several well-known drawbacks because of the higher temperature in the combustion chamber and the physical-chemical characteristics of diesel fuel, so that harmful pollutants are emitted, such as nitrogen oxides (NOx), particulate (PM), hydrocarbon compounds (HC), carbon monoxide (CO) and sulphur oxides (SOx). As reported by Park et al. [20], emissions of HC and CO are lower if DME is burned in a CI engine, whilst the absence of sulphur in DME fuel allows one to obtain SOx-free exhaust gases. Aiming to reduce the formation of particulate (soot), typical of diesel-fed CI engines, an anti-particulate filter (APF) should be used. The high oxygen content and the absence of C-C bonds in DME molecule does not favour formation of soot during combustion, eliminating the already described problem associated with diesel fuel. Experimental results reported by Sidhu et al. [21] showed that the relative particulate yield from DME was just 0.026% versus the value of 0.51% exhibited by both diesel and bio-diesel fuels. For this reason, an APF is not required in DME-fueled engines. Thanks to this advantage, the installation and application of oxidation catalysts for further reduction of both HC and CO is possible in terms of economy and vehicle space.

A trustworthy experimental comparison of NOx emissions from CI engines when using DME or diesel fuel is not easy to perform because the results strongly depend on the engine conditions and the fuel supply system. Usually, a higher NOx level was detected when diesel fuels were substituted with DME, as reported be Park et al. [20] and Kim et al. [22], but opposite results have been published in SAE International studies [23,24]. Unlike diesel fuel, the reduced emissions of the other pollutants allows one to use high exhaust gas recirculation (EGR), thus decreasing the NOx level without any increase in PM and soot emissions [19].

On the other hand, not only the combustion performance has to be taken into account when assessing the possibility of using a substance as an alternative fuel. In fact, a careful evaluation of the efficiency of each step, from the raw material supply to utilization of the final fuel is required. In this concern, a well-to-wheels (WTW) analysis is usually performed. A well-to-wheels analysis consists of a well-to-tank (WTT) and a tank-to wheels (TTW) analysis [19]. The WTT analysis can be carried out by calculating the WTT efficiency as the ratio between the energy of the fuel (e.g., in terms of lower heat value, LHV) and the sum of energy consumptions in each manufacturing step, from feedstock recovery to fuel distribution. Among the alternative fuels derived from natural gas, biomass or electrolysis (e.g., DME, methanol, synthetic diesel, hydrogen, etc.), DME exhibits the highest WTT efficiency [19]. TTW analysis includes everything related to the vehicle and its characteristics and for these reasons different fuels have to be compared under the same engine technology and, in this context, DME exhibits high engine efficiency for several vehicle technologies. By coupling WTT and TTW analysis, in order to estimate a WTW efficiency, Semelsberg et al. [19] according to Arcoumanis et al. [6], suggest that DME ranks on the top among different alternative fuels for several vehicle technologies. The WTW efficiency of DME is comparable with LPG and compressed natural gas (CNG)-fueled vehicles, but lower than vehicles operating with diesel fuel. 

Because of the clean emission offered during its combustion, DME is also suggested as fuel for power generation by using gas turbines. In the last decade, several companies (including BP, Snamprogetti/ENI S.p.A, Haldor Topsøe) [25] have tested DME as a gas turbine fuel in the case of reduced natural gas availability. As mentioned above, the physico-chemical properties of DME are close to those of LPG, which allows ocean transport using conventional LPG tankers without any additional effort. Several studies have demonstrated that DME is a clean fuel alternative to natural gas, in terms of both NOx and CO emissions [26,27]. Depending on the operation conditions, DME can emit more CO than NOx [28], but this disadvantage can be overcome by a slight nozzle modification [29].

As already discussed, hydrogen generation remains the major issue to boost the proposed CO_2_-based scenario, involving DME as energy vector [30,31]. Production of hydrogen by steam reforming of methane or gasoline is the main industrial process to produce hydrogen and these processes require high temperatures (above 600 °C for methane and above 800 °C for gasoline), high energy demand, stable catalysts and expensive insulated reactors. Recently, steam reforming of methanol is receiving attention because of the relative low process temperature (around 300 °C) and simpler reactor configurations [32]. Nevertheless, due to the high toxicity of methanol, DME is also considered a reliable candidate for hydrogen production by steam reforming (by adopting similar operation conditions of methanol steam reforming and over a bifunctional catalyst, namely an acid function for DME hydrolysis to methanol and a copper-based catalyst for the alcohol reforming), being not toxic and with a high hydrogen content [33,34,35,36]. 

## 3. Thermodynamic Considerations on CO_2_-to-DME Process

CO_2_ hydrogenation to DME usually involves four reactions summarized in Table 2.

For all of the involved reactions it is possible to calculate the equilibrium constants, as follows:

(1) $$K_{j}(T)=\prod_{i}a_{i}^{\nu_{ij}}=\exp\left(\frac{-\Delta {\tilde{G}}_{rj}}{RT}\right)$$

where *a_i_* is the activity of the specie *i* involved in the reaction *j* with the stoichiometric number *ν_ij_*, while $\Delta {\tilde{G}}_{rj}$ is the molar Gibbs energy change of the reaction *j* that can be calculated from the following equation:

(2) $$\Delta {\tilde{G}}_{rj}=\sum_{i}\nu_{ij}{\tilde{G}}_{i}(T,P)$$

Since all species are in gaseous phase a state standard activity of pure gas at 1 bar can be chosen as reference to compute activity:

(3) $$a_{i}=\frac{{\bar{f}}_{i}}{1\;\mathrm{bar}}$$

where ${\bar{f}}_{i}$ is the fugacity of the specie *i* in the gaseous mixture that has been computed adopting Peng-Robinson equation of state for the mixture.

Therefore, the equilibrium constants can be expressed as follows:

(4) $$K_{1}(T)=\frac{{\bar{f}}_{CH_{3}OH}\cdot {\bar{f}}_{H_{2}O}}{{\bar{f}}_{CO_{2}}\cdot {\bar{f}}_{H_{2}}^{2}}=\exp\left(\frac{-\Delta {\tilde{G}}_{r1}^{0}(T)}{RT}\right)$$

(5) $$K_{2}(T)=\frac{{\bar{f}}_{CH_{3}OCH_{3}}\cdot {\bar{f}}_{H_{2}O}}{{\bar{f}}_{CH_{3}OH}^{2}}=\exp\left(\frac{-\Delta {\tilde{G}}_{r2}^{0}(T)}{RT}\right)$$

(6) $$K_{3}(T)=\frac{{\bar{f}}_{CO}\cdot {\bar{f}}_{H_{2}O}}{{\bar{f}}_{CO_{2}}\cdot {\bar{f}}_{H_{2}}}=\exp\left(\frac{-\Delta {\tilde{G}}_{r3}^{0}(T)}{RT}\right)$$

(7) $$K_{4}(T)=\frac{{\bar{f}}_{CH_{3}OCH_{3}}}{{\bar{f}}_{CO_{2}}\cdot {\bar{f}}_{H_{2}}^{2}}=\exp\left(\frac{-\Delta {\tilde{G}}_{r4}^{0}(T)}{RT}\right)$$

Then, the mass balance on the specie *i* can be written as:

(8) $$n_{i}^{eq}=n_{i}^{0}+\sum_{j}\nu_{ij}\xi_{j}$$

where $n_{i}^{eq}$ and $n_{i}^{0}$ are the final and the initial moles of the specie *i*, while *ξ_j_* is the extent of the reaction *j*. 

Accordingly, the CO_2_ equilibrium conversion can be calculated as follows:

(9) $$X_{CO_{2}}=\frac{n_{CO_{2}}^{0}-n_{CO_{2}}^{eq}}{n_{CO_{2}}^{0}}=\frac{\xi_{1}+\xi_{3}}{n_{CO_{2}}^{0}}$$

Whilst selectivity towards DME, MeOH and CO, on C-basis, are defined according to the following equations:

(10) $$S_{DME}=\frac{2\cdot \xi_{2}}{\xi_{1}+\xi_{3}}$$

(11) $$S_{MeOH}=\frac{\xi_{1}+\xi_{4}-\xi_{2}}{\xi_{1}+\xi_{3}}$$

(12) $$S_{CO}=\frac{\xi_{3}-\xi_{4}}{\xi_{1}+\xi_{3}}$$

In this paragraph, the effect of reaction parameters, such as reaction temperature, reaction pressure and inlet H_2_/CO_2_ ratio, on thermodynamics of one-pot CO_2_ hydrogenation to DME are discussed. Simulation was performed using Unisim Design R430 software (Honeywell, Morristown, NJ, USA), by assuming only CO_2_ and H_2_ in the reactant mixture. 

**Figure 2:**
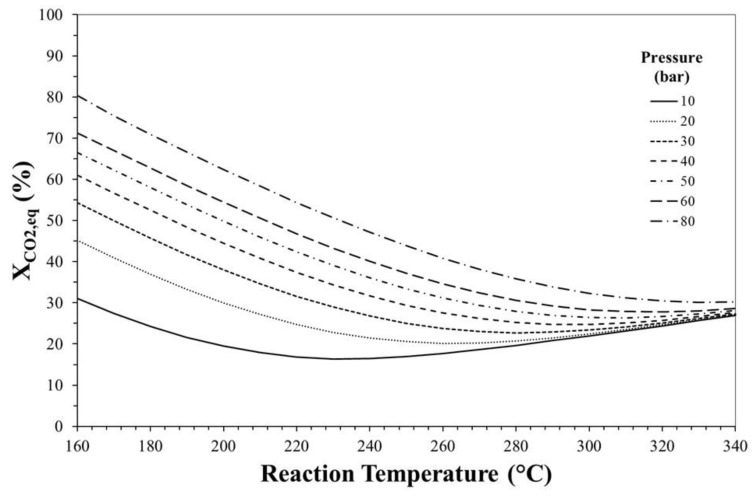
Effect of reaction temperature and pressure on CO_2_ equilibrium conversion. H_2_/CO_2_ (mol/mol) = 3.

Figure 2 reports the equilibrium theoretical conversion of CO_2_ as a function of reaction temperature and pressure, calculated for an initial H_2_/CO_2_ molar ratio equal to 3. 

The increase in reaction pressure promotes CO_2_ conversion since global reaction proceeds with a decrease in the number of moles even if this effect becomes less evident at high temperature. On the contrary, since the process involves both exothermic (i.e., step 1, 2 and 4 of Table 2) and endothermic (i.e., step 3) reactions, the effect of increase in temperature on CO_2_ equilibrium conversion is disadvantageous at low temperature and advantageous at high temperature, even if this effect is more marked at low pressure. For instance, at 10 bar, X_CO_2_,eq_ decreases from ca. 30% to ca. 16% when the temperature increases from 160 °C to 230 °C and it increases to ca. 28% if the reaction temperature rises to 340 °C, suggesting that at high temperature the reverse water gas shift reaction (that also favours CO_2_ consumption) predominates over the other steps. 

**Figure 3:**
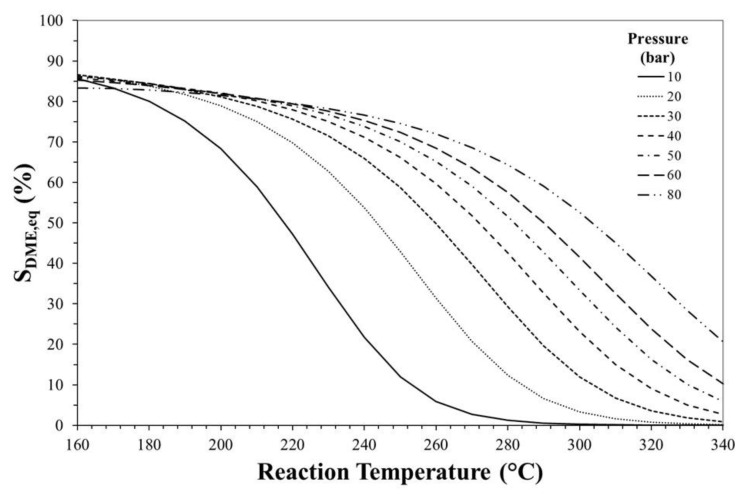
Effect of reaction temperature and pressure on DME equilibrium selectivity. Initial H_2_/CO_2_ molar ratio equals to 3.

**Figure 4:**
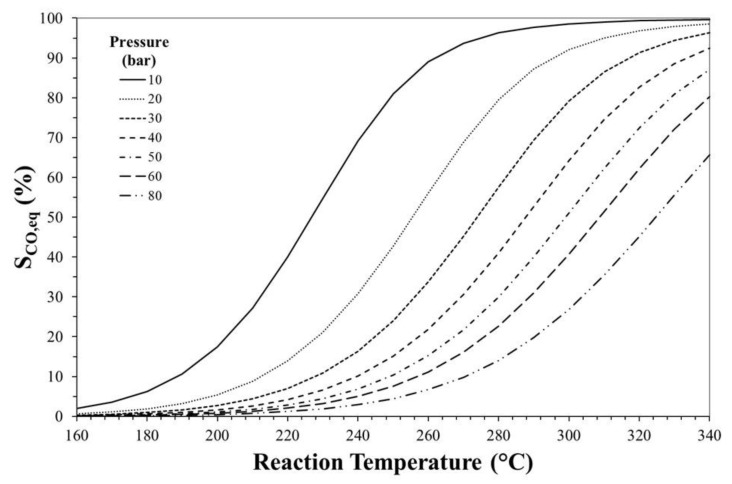
Effect of reaction temperature and pressure on CO equilibrium selectivity. Initial H_2_/CO_2_ molar ratio equals to 3.

**Figure 5:**
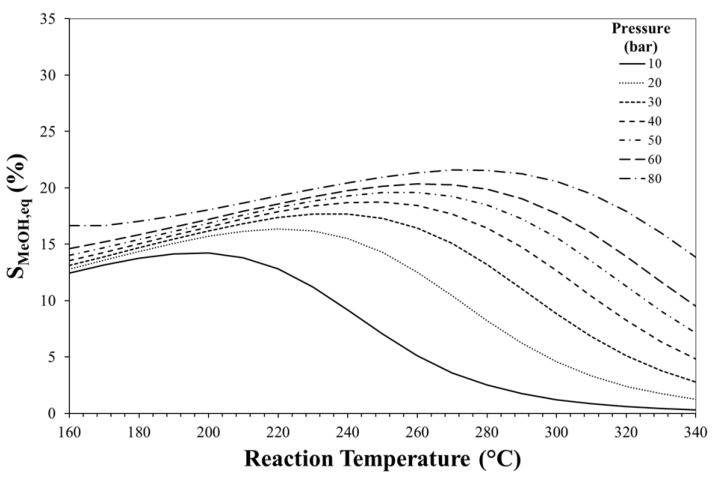
Effect of reaction temperature and pressure on methanol equilibrium selectivity. Initial H_2_/CO_2_ molar ratio equal to 3.

Figure 3, Figure 4 and Figure 5 show the effects of reaction temperature and pressure on selectivity towards DME, CO and methanol, respectively. As mentioned before, low temperature should be adopted for favouring exothermic reactions and achieving high DME selectivity. In fact, high temperatures favour rWGS promoting CO formation and lowering selectivity towards DME. 

Especially at high temperature, DME selectivity can be improved by increasing the reaction pressure. For instance, at 240 °C, DME equilibrium selectivity can be increased from ca. 20% to ca. 60% by pressuring the system from 10 bar to 30 bar, while selectivity towards CO can be reduced from ca. 70% to values below 20%.

**Figure 6:**
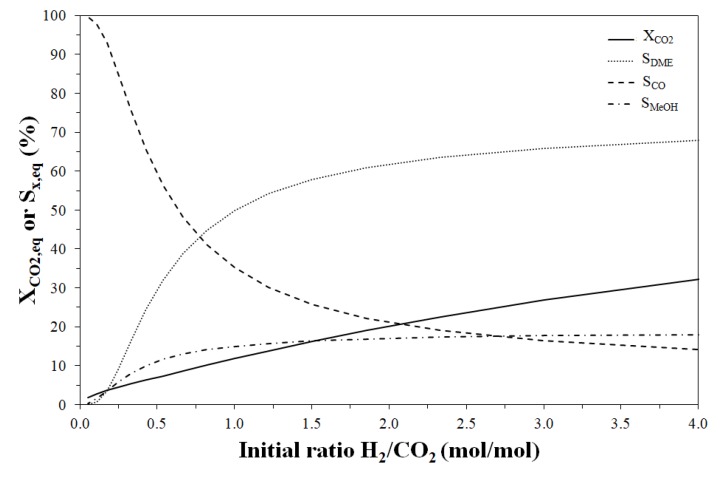
Effect of initial H_2_/CO_2_ molar ratio on CO_2_ equilibrium conversion and DME, CO and methanol equilibrium selectivity. Reaction temperature and pressure: 240 °C and 30 bar, respectively.

The equilibrium selectivity towards methanol is favoured by high pressure and low temperature even if it can be estimated always lower than 20%. Also the reactant mixture composition strongly affects process thermodynamics as shown in Figure 6.

Increasing the H_2_/CO_2_ molar ratio in the feedstock, the CO_2_ conversion also increases and a H_2_/CO_2_ value higher than 0.8 mol_H_2__/mol_CO_2__ (at 240 °C and 30 bar) should be adopted in order to obtain a selectivity to DME higher than that to CO. In fact, a higher CO_2_ concentration favours CO formation from rWGS causing a DME selectivity loss. On the contrary, higher DME selectivity values can be predicted for a higher H_2_ initial content, even if the effect is much more pronounced at low H_2_/CO_2_ ratio. 

### The Effect of Methanol Dehydration Reaction Step on Thermodynamics

**Figure 7:**
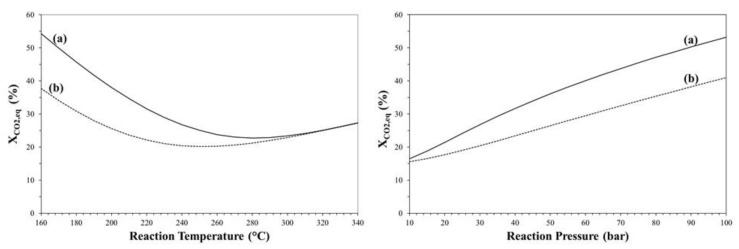
CO_2_ equilibrium conversion of CO_2_-to-DME (**a**) and CO_2_-to-MeOH (**b**) process as a function of reaction temperature (**left**) and pressure (**right**).

It is well known that DME production from CO_2_ can be carried out in a two-step process in which methanol is produced via CO_2_ hydrogenation in a first reactor, purified and then dehydrated to produce DME in another reactor, whilst in “one-pot” process both reaction steps are simultaneously carried out in the same reactor [37]. Figure 7 shows the effect of temperature and pressure on CO_2_ conversion for both one-step and two-step process.

Due to methanol consumption by dehydration reaction (2), the one-step process is more efficient than the two-step process in terms of CO_2_ equilibrium, even if such a thermodynamic benefit is more valuable at low temperature and high pressure. 

In order to quantify the thermodynamic benefit of using the one-pot process, the CO_2_ conversion gain (CPG) can be calculated as follows:

(13) $$CPG=\frac{X_{CO_{2},eq}^{a}-X_{CO_{2},eq}^{b}}{X_{CO_{2},eq}^{b}}\cdot 100$$

where $X_{CO_{2}}^{a}$ and $X_{CO_{2}}^{b}$ are the CO_2_ equilibrium conversion predicted for the one-step or the two-step process, respectively. 

**Figure 8:**
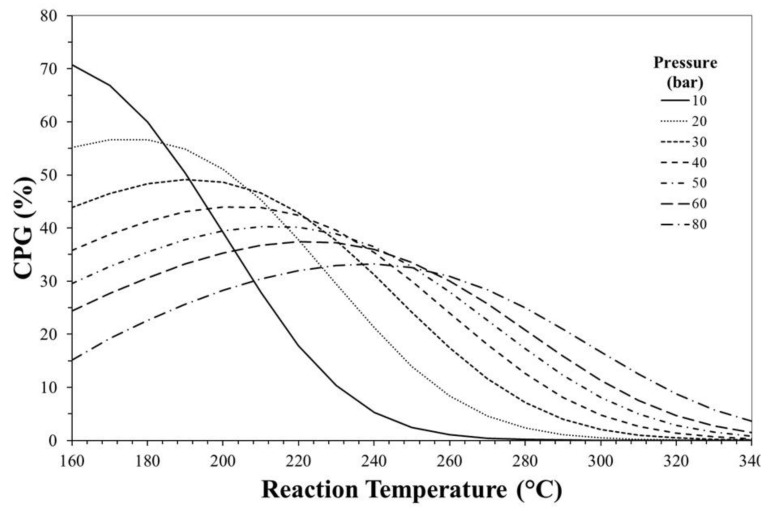
CO_2_ conversion percentage gain (CPG) as a function of reaction temperature and pressure.

Figure 8 shows CPG as a function of reaction temperature and pressure. The obtained graph can be used as a tool to individuate the optimal operation conditions maximizing the thermodynamic effect in terms of CO_2_ conversion. For instance, at reaction pressure of 30 bar, the maximum benefit in terms of X_CO_2__ to carry out the one-step rather than the two-step process can be obtained at ca. 200 °C. 

## 4. Catalytic Systems for DME Production

**Figure 9:**
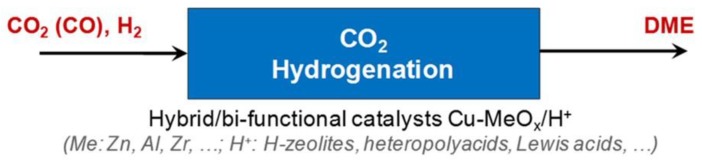
Scheme of the direct synthesis of DME through CO_2_ hydrogenation.

As before mentioned, gas phase DME synthesis could be performed in either a two-step process (indirect synthesis) or a single-step process (direct synthesis) [38]. In the conventional indirect synthesis, methanol is first synthesized over a metallic-based catalyst through COx hydrogenation [39], then methanol is dehydrated into DME over solid acid catalysts (such as γ-alumina, zeolite, heteropolyacids, …). On the other hand, in the direct DME synthesis the catalyst functionalities of methanol synthesis and in-situ dehydration are integrated in a bifunctional systems within a single reactor (see Figure 9) and this is an attractive alternative to the two-step process [40,41,42], also alleviating the thermodynamic constraints of methanol synthesis and leading to higher both CO_2_ conversion and DME selectivity [38,39,43]. Moreover, from an industrial point of view, the use of a single reactor should reduce the capital costs for the DME production [44,45,46].

It also noteworthy that the real direct DME synthesis product distribution may not correspond to the predicted values by thermodynamics as the product distribution are is also affected by reaction kinetic. Moreover a good understanding of the thermodynamic limitations for direct DME synthesis reaction can be useful for developing new chemical processes and improving the already existing-ones (see Section 3). Anyway, the indirect synthesis is the most diffused process for DME production [47] and also in this case, whatever the process, catalyst properties strongly affect process performances, such as product distribution and deactivation of the catalyst. In this paragraph, the effect of both metal or acid catalyst properties on DME production are discussed, emphasizing on the potential of zeolites as acid catalysts. 

### 4.1. Catalysts for CO_2_-to-MeOH Step

In the literature several catalytic systems are claimed as active in the activation of carbon dioxide. Table 3 lists the best performing catalysts, together with the active species used (Cu, Ag, Au, Ni, Pd, Pt), the preparation method and their respective physicochemical and morphological properties [47].

Among these formulations, copper-based catalysts exhibit the best catalytic performance in terms of CO_2_ hydrogenation to methanol. In addition, previous studies identified the following order of catalytic activity [48]:

Cu >> Co = Pd = Re > Ni > Fe >> Ru = Pt > Os > Ir = Ag = Rh > Au

On the whole, this reactivity trend confirms that copper-based systems are the most active ones in the CO_2_ activation. In any case, it must be pointed out that catalytic activity strongly depends on metal dispersion, the use of dopants, and the choice of the support. A further key point regarding catalytic hydrogenation from CO_2_ rather than syngas is related to the higher oxidation power of CO_2_ with respect to CO, thus affecting the active state of the catalyst for methanol synthesis and consequently the methanol formation rate [49]. Therefore, there is a strong influence of the reaction conditions on the overall catalytic behaviour and the need to develop appropriate kinetic models to describe the overall synthesis. This is the basis for a proper modelling of the process and its optimization.

The first commercial catalyst to convert syngas to methanol was produced by BASF (Ludwigshafen, Germany) in 1923 using ZnO-Cr_2_O_3_ catalyst, active at high temperature (350–400 °C) and pressure (240–350 bar) [44]. However, this catalyst was easily poisoned by impurities of the feed syngas such as sulfur, chlorine and heavy metals. Imperial Chemical Industry (ICI) introduced a more active and stable Cu/ZnO based catalyst in 1966 [39]. However, although strongly influenced by operating conditions and the preparation methodology, a careful literature data analysis showed that the best systems for the hydrogenation of CO_2_ to methanol are Cu/ZrO_2_ based [50]. In fact, overcoming the empirical approach, in catalyst design detailed knowledge of the espected catalytic properties is requested, particularly about the control of morphological properties (e.g., total surface area, metal dispersion, crystallinity). This objective can be achieved through a fine design of the catalytic system, adopting a suitable preparation method.

Although the Cu-based systems supported on ZnO have been the most studied catalysts for CO_2_ hydrogenation, it is clear that the specific activity of copper does not seem apparently influenced by the nature of the carrier oxide. However, previous studies conducted by Chinchen et al. [51] showed that the Zn exerts many functions, giving the best performance among the different tested oxides (Cr_2_O_3_, SiO_2_ and MnO). Zinc oxide acts as a geometrical spacer between Cu nanoparticles and thus plays a pivotal role in maintaining the active Cu metal in optimal dispersion in the Cu/ZnO catalyst, consequently providing a high number of active sites exposed to gaseous reactants [43]. Indeed, beyond to promote the increase of the surface area (especially with alumina), ZnO is enough refractory and it hinders the sintering of copper particles, acting as a dispersing agent of sulphur and chlorides as well, the main poisons for the catalyst. ZnO also plays an important role to maintain an appropriate ratio Cu^+^/Cu^0^, since both states are involved in the synthesis, creating Cu^+^-O-Zn type active sites and thus stabilizing the oxidation state of copper. Behrens et al. [42] have reported that ZnO can promote strong metal–support interaction with Cu species, which induces the formation of “methanol-active copper”. Nakamura et al. [52] found that Zn shows a very good promoting effect in the synthesis of methanol, but not in rWGS reaction. Furthermore, addition of trivalent ions like Al^3+^ into the Cu/ZnO was found to improve stability along with Cu dispersion and metal surface area. Afterwards, a ternary Cu/ZnO/Al_2_O_3_ (CZA) catalyst started to be used for methanol synthesis operating at moderate pressure ranging from 50 to 100 bar and temperature of ca. 250 °C [37,38,39,40,41,42].

Although it is unanimously recognized the peculiar functionality both of copper and zinc oxide in the mechanism of CO_2_ activation, for a further development of the catalytic system, the need for other metal oxides into the catalyst composition is also required, so to realize multimetallic systems more active than bimetallic catalysts in the formation of MeOH, which will be then dehydrated into DME. Really, many studies have already reported the unique features of various metals added in the catalyst composition as promoters of Cu-Zn based catalysts for the CO_2_-to-MeOH hydrogenation reaction, like Al [53,54,55,56], Mn [57,58], Cr [59], Au [60], Zr [61,62,63,64,65,66,67,68], Pd [69,70,71], La [55,72,73], Si [74,75], Ce [55,76], Ga [77,78], V [79], carbon [80,81,82] or mixtures among them [55,83,84,85,86] revealing a superior performance of Zr, Al and Ga in terms of activity, selectivity and stability.

### 4.2. Catalysts for MeOH-to-DME Step

Dimethyl ether is produced via methanol condensation/dehydration (MeOH-to-DME, MTD):

2CH_3_OH = CH_3_OCH_3_ + H_2_O

As discussed above, methanol dehydration is an exothermic reversible reaction that proceeds without mole number variation. For this reason, reaction pressure does not affect conversion equilibrium, while lower reaction temperatures have a thermodynamic benefit toward DME production. Methanol dehydration is an acid-catalyzed reaction and several investigations have been published with the aim to identify an active, selective and stable catalyst at relative low temperature for the above-mentioned thermodynamic advantages. Furthermore, for this technology there are several licensors including Haldor Tospoe, Linde/Lurgi, Toyo Engineering, Uhde, MGC (Mitsubishi Gas Chemical Company, Tokyo, Japan), China Southwestern Research Institute of Chemical Industry and China Energy (Jiutai Group, Shanghai, China). 

Depending on catalyst characteristics, methanol dehydration can be carried out in both vapour and liquid phase, with reaction temperature in the range 100–300 °C and pressure up to 20 bar, being γ-Al_2_O_3_ the most investigated solid acid catalyst, due to its low cost, high surface area, good thermal and mechanical stability. Furthermore, γ-Al_2_O_3_ shows high selectivity to DME even at high temperature (up to 400 °C) thanks to the presence of weak Lewis acid sites not able to promote side reactions. Unfortunately, these acid characteristics require reaction temperature higher than 250 °C to favour high methanol conversion [85,86], but catalyst activity can be improved by modifying γ-Al_2_O_3_ surface with silica, aluminium-phosphates, titanium, niobium, boron or others species [87,88,89,90,91]. 

As demonstrated by several investigations [88,92,93,94,95], despite γ-Al_2_O_3_ offers high selectivity towards DME, it tends to strongly adsorb water produced during the reaction causing deactivation. As above described, an important amount of water is produced especially in the one-pot CO_2_-to-DME process. Thus, γ-Al_2_O_3_, in spite of its several advantages, is not classified as a reliable acid catalyst for DME production by CO_2_ hydrogenation.

Heteropolyacids (HPAs) can be also used to catalyze the methanol dehydration step. HPA-type materials can be represented by the formula H_8−n_[X_n_^+^M_12_O_40_], where “X” is the central atom (e.g., P^5+^, Si^4+^, Al^3+^, etc.), “n” is its oxidation state and “M” is the metal ion. Because the high Brønsted acidity displayed from these materials, HPAs offer catalytic performances usually better than other solid catalysts such as zeolites, e.g., ZSM-5, especially at low temperature. Alharbi et al. [96] compared HPAs catalysts with ZSM-5 zeolites with different acidity (Si/Al = 10–120). Results showed that tungsten/phosphorous-containing HPA (HPW) showed a turnover frequency (TOF) of about 53 h^−1^, while the most active ZSM-5 sample showed a TOF value of about 1 h^−1^. The authors highlighted that the superior catalytic activity displayed by HPAs can be attributed to a higher acid site strength of these materials. Furthermore, Ladera et al. [97] reported that the accessibility of methanol molecules to proton sites of HPAs can be improved by using TiO_2_ as support. 

Ion exchange resins (IERs) were also proposed as acid catalysts for methanol dehydration. Divinil benzene/styrene copolymers are usually used in which sulfonic acid groups are introduced being able to dehydrate methanol to DME. IERs have been considered as attractive catalysts for MTD reaction because the high activity exhibited even at relatively low temperature (<150 °C) [98]. Recent works suggested Nafion resin, Nafion/silica composites or Amberlyst 35/36 as suitable catalysts for the synthesis of DME from methanol [99,100,101]. On the other hand, the application of ion exchange resins as acid catalysts in the one-pot CO_2_-to-DME hydrogenation is hindered by the low thermal stability usually exhibited from these materials under the typical process temperature range (ca. 250 °C) [102].

#### Methanol Dehydration over Zeolites

Research is now focusing on use of zeolites as catalysts for the methanol dehydration step, especially in view of a potential application as acid catalyst for the one-pot CO_2_-to-DME process. Zeolites are applied also in other catalytic processes concerning the reuse of carbon dioxide and production of alternative fuels, such as dry reforming of methane [103,104,105] and biodiesel production by enzymatic catalysis [106,107].

Zeolites are crystalline aluminosilicates whose catalytic properties are well-known for decades. The ever-growing application of zeolites as catalysts in several industrial processes is mainly due to their unique molecular shape-selectivity resulting from a well-defined regular microporous structure. The possibility of tuning this system of voids (openings and spatial orientation of channels, size and location of cages, etc.) allows to have a catalyst that is able to catalyze the specific reaction pathway. Beside to shape-selectivity, the acid properties of zeolites are of paramount importance in catalysis. Generally, both Brønsted and Lewis type acid sites are simultaneously present in zeolites and their concentration, distribution, strength and location are well known factors affecting the overall activity, product selectivity and deactivation of the catalyst. Such aspects and further insights about the use of zeolites in catalysis are fully reported by Corma [108]. 

Several investigations [85,109] have been already focused on use of ZSM-5 catalyst for methanol dehydration reaction, exhibiting, unlike γ-Al_2_O_3_, high resistance toward water adsorption. Furthermore, due to its stronger acid sites (Lewis and/or Brønsted type), ZSM-5 offers high activity in terms of methanol conversion at a relatively low reaction temperature. For instance, Vishwanathan et al. [109] reported a value of methanol conversion of about 80% over H-ZSM-5 at 230 °C, while over γ-Al_2_O_3_ conversion was just 5%, being necessary to increase the temperature to 320 °C to reach 80%, thus affecting both the process economy and the thermodynamic gain. High activity is also retained because ZSM-5 possesses medium and strong acid sites that allow a fast methanol conversion. Unfortunately, as above mentioned, strong acid sites of zeolites catalyse also other methanol-involving side reactions, leading to the formation of by-products such as olefins and coke, definitely causing a loss of DME selectivity and deactivation. 

The mechanism of DME formation over zeolites by methanol dehydration has been already investigated [110,111,112,113], demonstrating that it involves the formation of methoxyl ions by reaction among methanol and the acid sites of the catalyst, followed by combination of another alcohol molecule with methoxy species to form DME (even if the associative mechanism involving two methanol molecules co-adsorbed on the same acid site cannot be excluded). On the contrary, in the temperature range of the direct synthesis of DME (250–280 °C), the strong acid sites of zeolite may convert the methanol into a wide range of hydrocarbons: from olefins (methanol-to-olefins process, MTO) [114,115] to aromatic species that are somehow linked to the olefins production, being involved in the (auto)-catalytic process known as “hydrocarbon pool” mechanism (HCP) [111,115,116,117]. Under the simultaneous presence of aromatic compounds, acid sites and high temperature, a favourable condition is realized for coke formation, a relevant aspect in MTO. Catalyst structure (channel size and configuration) is the most important factor addressing the pool-molecules formation. At high temperature, characteristic of MTO process, zeolite structures with large cages or 3-D channel system, as SAPO-34, BEA and MFI, promote the formation of aromatic compounds that can be entrapped in the structure or diffuse out. The most common aromatic species are in the class of poly-methylbenzenes, depending on the catalyst channel size: tri-methylbenzene is the most active species on MFI, while hexa-methylbenzene is the most active for olefins formation in the large cages of SAPO-34 and BEA [118]. These molecules are also considered as coke precursors and it is important to notice that they can be produced also in the lowest temperature range of MeOH-to-DME reaction. Under such conditions these poly-methylbenzenes do not act as co-catalysts in HCP (because of the low temperature), accumulating in the structure as carbon deposits. Furthermore, in the case of either DME or olefins synthesis, it is well known that zeolite deactivation mainly comes from the coke deposition inducing pore blocking. In addition, it has been demonstrated that both catalyst structure and acidity strongly affect the mechanism of coke formation, in terms of composition, quantity and location. Campelo et al. [119] reported a comparison between several silico-aluminophosphates with different channel configuration (1-, 2- and 3-dimension), showing that on a 3-dimensional structure (as SAPO-34), the oligomers formed in the channels can migrate to the big cages of this structure, where react over strong acid sites so leading to the formation of heavier oligomers and aromatics that cannot back to the channels, causing a rapid catalyst deactivation for pore blocking. On the other hand, deactivation of 1-dimensional large channels (as SAPO-5) is due to the adsorption of multi-branched chains on the strong acid sites, also causing blocking of the pore system. Structures with both small/medium channels and cages, as MFI type, do not permit trapping of heavy compounds inside the crystal and coke is preferably formed on the external surface of crystals, so that catalyst deactivation occurs by coke deposition on the mouth of the channels [120,121]. Catalyst deactivation rate is also affected by crystal morphology: small or hierarchical crystals exhibit higher resistance to deactivation by coke deposition than large crystals with microporous texture [122,123]. On small 1-D structures, as MTF, no hydrocarbon pool mechanism is observed even at high temperature (400 °C) and DME is the only product detected in reactor out-stream; nevertheless this structure exhibits fast deactivation over time [124] as consequence of carbon deposition. Therefore, catalyst structure may play a key role on product selectivity, inhibiting either the hydrocarbon pool mechanism or the formation of coke precursors with the aim to increase the catalyst stability overtime.

Recently, several studies were dedicated to study the effect of zeolites structure on catalytic performances during methanol-to-DME reaction step at reaction temperature of direct synthesis (<280 °C). 

On 2017, Catizzone et al. [125] studied methanol dehydration reaction on several zeolites (or molecular sieves) having different channel orientation (1-, 2- and 3-dimensional channel orientation) and channel openings (from 8- to 12-membered rings). Results clearly showed how the voids system of zeolites is of paramount importance for this reaction. 1-dimensional zeolites with large pore openings (e.g., MTW or MOR) showed high selectivity towards DME (at 240 °C), but a fast deactivation rate was observed, attributable to pore blocking by coke deposition. On the contrary, by using 1-dimensional zeolite with medium-pore openings (such as TON), both a higher stability towards deactivation and lower coke deposition was observed. Zeolites with 3-dimensional channel orientation (i.e., MFI, BEA or SAPO-34) were also investigated by the authors [125,126]. Despite the small channel openings of SAPO-34 (about 3.8 Å) conferring high selectivity towards DME, this catalyst was rapidly deactivated by coke formed and deposited into the large 3-dimensional channel intersections (about 7.4 Å large), so preventing reactant diffusion inside the crystal. On the contrary, when channel openings and intersections have similar size (e.g., in the case of BEA or MFI structure) a higher stability was observed, even if by-products (e.g., C_2_^=^–C_6_^=^) were detected in the reaction out-stream and a high coke deposition rate was observed [127].

Coke deposited during methanol dehydration to DME over zeolites mainly consists of poly-methylbenzenes (PMB, ranging from xylenes to hexamethylbenzene) with a grade of substitution that is a function of the channel system. For instance, the 1-D medium pore channel system of TON inhibits the formation of PMB heavier than tri-methyl benzene; on the contrary, despite the similar channel openings of TON, EUO-type structure accommodates also hexa-methyl benzene due to the presence of side-pockets large enough to permit the formation of this molecule. Zeolites with large pore system, such as beta, also allows deposition of polycyclic species, while carbon phase deposited on FER-type crystals selectively consists of tetramethylbenzene compound probably located in ferrierite cages [125].

Coke formation can be reduced by post-synthesis treatment or by a careful tailoring of the textural properties. A dual pore size distribution (e.g., micro- and mesopores) is a reliable configuration to reduce coke formation and postpone catalyst deactivation during methanol dehydration. Tang et al. [128] reported that a ZSM-5/MCM-41 composite material with both microporous and mesoporous allows to obtain higher activity and higher stability than ZSM-5 with only microporous structure. Similar results were obtained by Rutkowska et al. [129] which showed that hierarchical ZSM-5 material (interconnected micro-/mesopores) exhibits a lower coke formation rate than microporous ZSM-5.

On the whole, several results suggest FER zeolite as a reliable catalyst to selectively transform methanol to DME [125,130,131]. Thanks to its 8-/10-membered ring 2-dimensional channel system, FER structure shows a high DME selectivity, a slow coke deposition and a high resistance to deactivate. 

Beside the structure, the acidity properties of zeolites also strongly affect the catalytic behaviour of these materials. High catalytic performances, in terms of DME selectivity, were obtained by decreasing the acid site strength, upon modification of the zeolite surface [132,133], or by decreasing total acidity [109,134,135].

Kim et al. [132] studied methanol dehydration reactions over ZSM-5 zeolites impregnated with different γ-Al_2_O_3_ loading and compared the catalysts in terms of operative temperature range (OTR), defined as the temperature range giving methanol conversion higher than 50% and DME selectivity higher than 99%. The authors found that OTR of 210–310 °C and 320–370 °C were evaluated for bare ZSM-5 and γ-Al_2_O_3_ confirming that the zeolite is more active but less selective than γ-Al_2_O_3_. On the contrary, a hybrid catalyst γ-Al_2_O_3_/ZSM-5, containing 70% γ-Al_2_O_3_, exhibited an OTR of 230–380 °C showing, therefore, higher activity than γ-Al_2_O_3_, but also higher selectivity than both γ-Al_2_O_3_ and bare ZSM-5 thanks to the suitable dilution of strong acid sites of zeolite after impregnation.

Similar results were obtained by other authors by impregnation of ZSM-5 catalyst with sodium, magnesium, zinc or zirconium [133,134]. The authors showed that metal loading decreases strong acidity and increases weak acidity, forming a catalyst less active towards hydrocarbon formation. For instance, Khandan et al. [134] reported that DME yield increased from 53% to 93% after impregnation of ZSM-5 with zirconium; moreover, stability tests showed that resistance towards deactivation was also improved.

Acidity in zeolites can be also decreased by reducing the aluminium content or increasing the Si/Al ratio [135,136]. In this concern, Hassanpour et al. [135] prepared ZSM-5 catalysts in a wide range of Si/Al ratio (Si/Al = 25–250) and tested them in methanol conversion at 300 °C. The authors found that by-products formation (e.g., ethylene, propylene) decreases as the Si/Al increases, obtaining higher DME selectivity for catalysts with lower total acidity.

Catizzone et al. [131] have recently reported some aspects concerning the role of acid sites of FER-type zeolites by preparing catalysts with different Si/Al ratio. The authors reported that higher methanol conversion levels can be achieved over FER zeolites with higher aluminum content, but experimental evidences also demonstrated that lattice Lewis acid sites are more active than Brønsted sites even if a reaction temperature lower than 260–280 °C should be adopted in order to prevent by-products formation (especially methane). As previously discussed about thermodynamic aspects, a good catalyst for methanol dehydration step should promote high methanol conversion at temperature lower than 240–260 °C. The superiority of FER zeolite over γ-Al_2_O_3_ and several other zeolites at temperature lower than 240 °C was demonstrated recently [125]. In this paper, the authors showed that, at only 200 °C, methanol conversion was about 82% over FER with a 100% of DME selectivity, while a methanol conversion level of 25% only was observed for commercial γ-Al_2_O_3_. As discussed below, recent studies consider FER-type zeolite as a very promising acid functionality for preparing an attractive hybrid catalyst for CO_2_ hydrogenation to DME.

### 4.3. Catalysts for One-Pot CO_2_ Hydrogenation to DME

The catalyst for the direct CO_2_-to-DME conversion should be able to efficiently catalyze both methanol synthesis and its dehydration, minimizing the yield of CO formed by the Reverse Water Gas Shift (rWGS) side reaction and possible hydrocarbons from methanol conversion. A huge amount of water formed during CO_2_ hydrogenation to DME thermodynamically limits both formation and dehydration of methanol, causing a DME yield lower than that obtained via CO hydrogenation [85]. 

In this concern, the acid catalyst must be stable in presence of water and the acid sites must be well distributed and not too strong in order to inhibit hydrocarbons formation [85,89,137,138,139,140,141,142,143,144]. In this sense, as above described, zeolites seem to offer the highest versatility in terms of higher number of acid sites, water resistance and shape-selectivity towards the desired compound. A list of some results [77,145,146,147,148,149,150,151,152,153,154,155,156,157] concerning the one-pot CO_2_ hydrogenation to DME is reported in Table 4.

The first studies on hybrid/bifunctional catalytic systems active in the direct hydrogenation reaction of CO_2_ dealt with the use of physical mixtures between a methanol synthesis catalyst (MSC) and an acid system, typically a Cu-ZnO-Al_2_O_3_ system for the synthesis of MeOH and γ-Al_2_O_3_ or zeolites as acid solids for the dehydration of MeOH (see Section 4.2).

Zeolites, in particular, have shown greater efficiency than γ-Al_2_O_3_ as acid components of the bifunctional catalyst, considering that the possibility of modulating acidity (in terms of number, type and strength of acid sites) enables direct synthesis of DME at low temperatures, where the formation of methanol is thermodynamically favored. For instance, Naik et al. [145] compared the catalytic activity of hybrid catalysts prepared by mechanical mixing of MSC and γ-Al_2_O_3_ or ZSM-5 (Si/Al = 60). Catalytic tests carried out in a fixed-bed reactor at 260 °C and 5 MPa revealed that MSC/ZSM-5 exhibits a superior catalytic behaviour than MSC/γ-Al_2_O_3_ in terms of CO_2_ conversion (ca. 30% vs. ca. 20%), DME selectivity (ca. 75% vs. ca. 5%) and stability. As a conclusion, DME yield was about 20% over MSC/ZSM-5 and lower than 1% over MSC/γ-Al_2_O_3_ suggesting that γ-Al_2_O_3_ cannot be considered as a valuable acid catalyst for the one-pot CO_2_-to-DME process.

Compared to the conventional mechanical mixing between a methanol synthesis catalyst and a zeolite, the generation of metal oxide and acid sites in a single system is capable of improving the conversion of CO_2_, enabling even higher rates of formation/dehydration of methanol on the neighbouring surface sites. Different strategies have been applied to prepare the bifunctional catalysts for the direct DME synthesis. For instance, methods including impregnation, co-precipitation [158,159,160,161,162] or sol-gel steps (or their combinations) [44,163] and even more sophisticated approaches leading to core–shell catalyst structures [164,165,166] have been reported. Nevertheless, there are different opinions on the efficiency of bifunctional catalysts in comparison to admixed systems. Sun et al. prepared bifunctional catalysts by coprecipitation-sedimentation of a ZrO_2_-doped CZA-component on an H-ZSM5 zeolite [152,167]. According to that, bifunctional catalysts lead to high CO-conversions because the two types of active sites are in close contact with each other. This enables the generation of DME directly from an adsorbed methoxy-species without the intermediary synthesis of methanol.

Ge et al. also concluded that both active sites need to be in close contact so that a synergistic effect can be achieved, which leads to higher catalytic activity [44]. However, Ge et al. stressed that coverage of both active sites during catalyst synthesis needs to be avoided.

A coverage of the active sites leads to a drop of activity due to the synthesis of inactive species and due to a decrease in active surface area. García-Trenco et al. stated that admixed catalyst systems are superior to bifunctional systems [168]. Therefore, the preparation procedure of bifunctional catalysts can lead to a decrease of surface area due to a pores blockage. However, it is also stated that further interactions between the two active components, which have not been elucidated yet, might influence catalyst activity as well. 

Although the design of the catalytic system ideal for the hydrogenation of CO_2_ does not require properties such as selectivity of shape and/or size, due to the small size of the molecules involved, the zeolites still have a high potential expressed through multiple properties, such as tunable acidity, high surface area, microporosity or supporting properties, which represent the fundamental reasons featuring these unique structures as metal and/or metal-oxide carriers in most of current catalytic systems. 

Frusteri et al. [161] have recently investigated the role of acid sites of hybrid catalysts prepared via gel-oxalate precipitation of CuZnZr precursors of ZSM-5 crystals with a Si/Al ratio in the range 27–127. Results displayed that acidity of zeolite must be carefully tuned aiming at achieving a compromise between catalytic activity and resistance to deactivation by water. In particular, ZSM-5 with high Si/Al ratio (e.g., 127) showed high resistance in presence of water but low capacity to dehydrate methanol, while more acidic samples (e.g., ZSM-5 with Si/Al ratio of 27) offers high methanol conversion but poor water resistance and low DME selectivity. The authors found that, for the ZSM-5 zeolite, a Si/Al ratio of 38 showed the best performance in terms of CO_2_ conversion, DME yield and water resistance. 

Beside acidity, the textural properties of zeolite crystals also strongly affect the catalytic behaviour of hybrid catalysts. In this regard, the one-pot CO_2_-to-DME reaction in the presence of hybrid grains prepared via co-precipitation of CuZnZr precursors over zeolite crystals with different channel systems (i.e., MOR, FER and MFI) was recently investigated [158]. The microscopy analysis of hybrid grains revealed that zeolite crystal features strongly affect the metal-oxide(s) distribution, producing a very homogenous distribution over lamellar FER-type crystals and a formation of metal clusters on the other zeolite crystals. Catalytic results revealed that DME productivity followed the trend: CuZnZr/FER > CuZnZr/MOR > CuZnZr/MFI, and the authors related the superior catalytic activity of FER-based catalyst to lower mass transfer limitations offered by anchorage of metal-oxide clusters on the lamellar crystals typical of FER zeolite as well as to a larger population of Lewis basic sites generated on FER surface able to activate carbon dioxide and promoting its conversion. Nevertheless, an important deactivation of the catalyst was observed during time-on-stream tests. 

The hybrid catalysts developed so far generally tend to suffer deactivation by either coke deposition or metal sintering or poisoning from contaminants present in the reaction stream leading to the blockage of active sites [169,170]. As before mentioned, for hydrocarbon reactions over zeolites, deactivation is mainly attributed to two main mechanisms: (1) acid site coverage which deactivates the catalyst by coke adsorption; (2) pore blockage due to deposition of carbonaceous compounds in cavities or channel intersections that makes pores inaccessible [171]. In addition, it is well known that coke formation on zeolites is a shape-selective process. Under comparable conditions, large-pore zeolites are more susceptible to deactivation by coke deposition than medium-pore zeolites [172]. Although H-ZSM-5 is not sensitive to water [173,174], it shows high activity for the transformation of DME into hydrocarbon byproducts. These hydrocarbons can further evolve into heavy structures (coke) and consequently can block the zeolite pores and cause its deactivation, as previously discussed. However, this deactivation is slow due to the high partial pressure of hydrogen that attenuates the mechanism of coke formation [148]. This phenomenon can be controlled by employing a suitable concentration of Na in the zeolite in order to moderate the number of Brønsted sites and to reduce the acid strength of the H-ZSM-5 zeolite [170]. The addition of Silicalite-1 shell to the ZSM-5 zeolite is also considered as an efficient method for improving the resistance to the carbon formation [174]. As a rule, the reactor configuration strongly affects catalyst deactivation: considering that the reactors mostly used in the production of DME are slurry or fixed bed reactors, the bifunctional catalysts were seen to deactivate more quickly in slurry rather than in fixed-bed reactors [175]. 

These examples show that a deeper understanding of the underlying mechanisms and interactions among the active components both in admixed and in bifunctional catalysts is necessary. Furthermore, the influence of the catalyst composition as well as the main features controlling catalytic activity and stability should be studied in more detail, so that a comparison of the catalyst performance independent of the adopted preparation method will be possible.

## 5. Conclusions and Perspectives on the Catalyst Development

Zeolites are unique materials with huge catalytic potential in several industrial processes, recently generating great research interest as dehydration components in the synthesis of DME, starting not only from methanol, but even from CO_2_-rich streams recycled for hydrogenation. From the analysis of the thermodynamic equilibriums involved in the hydrogenation of CO_2_, it has been demonstrated that the production of DME is favoured at high pressure and low temperature for achieving both high conversion and DME yield. During CO_2_-to-DME hydrogenation reaction (P ≥ 30 bar; T ≤ 260 °C) the use of zeolites does not imply to simply exploit their typical shape/size selectivity, but other important features are involved, such as tunable solid acidity, high surface area, microporosity or loading property, representing the fundamental reasons why these unique structures are utilized as carriers of metals and/or metal-oxides.

Generally, a mechanical mixture of mixed oxides (containing Cu as active species for the synthesis of methanol) and a zeolite, typically ZSM-5, have been mainly proposed as an effective catalytic system. However, recent papers have claimed alternative zeolite structures as more suitable for the process, evidencing how the zeolite topology significantly affects the physicochemical properties of the catalysts as well as their catalytic performance. In particular, some fundamental aspects have been indicated as crucial for high process productivity: (i) the zeolite must be stable in presence of water; (ii) the formation of olefins should be inhibited; (iii) the acid sites must be well distributed and of suitable strength. Moreover, the performance of multi-site systems for the direct conversion of CO_2_ into DME has been also proved, so demonstrating the possibility to integrate the two methanol-synthesis and methanol-dehydration functionalities at level of single grain during preparation. Apart from the need of optimal experimental parameters, the crucial issue for preparing a high-active hybrid catalyst is optimizing the formulation and interaction of the different metallic, oxide(s) and acidic components, so to realize a punctual mix of surface sites necessary for the primary formation of methanol, followed by its dehydration to DME on the acid sites of the zeolite. Overall, in the perspective to develop an active and stable multi-site catalyst for DME production via CO_2_ hydrogenation, the concurrence of textural, structural and surface factors must be adequately balanced.

## Figures and Tables

**Table 1:** Physicochemical properties of DME and diesel fuels [6].

Property	Unit	DME	Diesel
Carbon content	mass%	52.2	86
Hydrogen content	mass%	1–3	14
Oxygen content	mass%	34.8	0
Carbon-to-hydrogen ratio	-	0.337	0.516
Liquid density	kg/m^3^	667	831
Cetane number	-	>55	40–50
Autoignition temperature	K	508	523
Stoichiometric air/fuel mass ratio	-	9.6	14.6
Normal boiling point	K	248.1	450–643
Enthalpy of vaporization	kJ/kg	467.1	300
Lower heating value	MJ/kg	27.6	42.5
Ignition limits	vol% in air	3.4/18.6	0.6/6.5
Elastic Modulus	N/m^2^	6.37 × 10^8^	14.86 × 10^8^
Liquid kinematic viscosity	cSt	<0.1	3
Surface tension (at 298 K)	N/m	0.012	0.027
Vapour pressure (at 298 K)	kPa	530	<10

**Table 2:** List of reactions involved in one-pot CO_2_ hydrogenation to DME.

Reaction No.	Reaction Stoichiometry	$\Delta \tilde{H}{{}^{\circ}}_{298\;K}$
1	CO_2_ + 3H_2_ = CH_3_OH + H_2_O	−49.5 kJ/mol_CO_2__
2	2CH_3_OH = CH_3_OCH_3_ + H_2_O	−23.4 kJ/mol_DME_
3	CO_2_ + H_2_ = CO + H_2_O	+41.2 kJ/mol_CO_2__
4	CO + 2H_2_ = CH_3_OH	−90.6 kJ/mol_CO_

**Table 3:** Textural and catalytic properties of metal/zirconia catalysts (Reproduced from [47] with permission of The Royal Society of Chemistry, license number: 4254680877107, 23 December 2017). Numbers in the last column refer to references number of reported in Ref. [47].

Catalyst M/ZrO_2_	Prep. ^a^	Precursors	BET Surface (m^2^∙g^−1^)	Metal Surface (m^2^∙g^−1^)	Product Selectivity ^b^			Ref.
					CH_3_OH	CO	CH_4_	
Cu	impreg.	Copper formate	-	-	-	-	-	11-13
Cu	impreg.	Copper tetrammine	107	1.8	35	65	0	14
Cu	co-prec.	Nitrates	64	-	68	32	0	15,16
Cu	co-prec.	Nitrates	174	7.1	66	34	0	14,17
Cu	co-prec.	Chloride/Sulfate	48.4	-	53	47	0	18
Cu	Ho-prec.	Nitrates	161	3.0	69	31	0	17
Cu	Prec.	Nitrates	86	-	15	57	28	19
Cu	Sol-gel	Acetate	215	-	40	60	0	20
Cu	alloy	Cu_70_Zr_30_	47	5.0	64	36	0	21
HT-Cu	Sol-gel, 2	Acetate	128	0.8	100-55			22
HT-Cu	Sol-gel, 1	Acetate	100	-	100-55			22
HT-Cu	Sol-gel, 1	HNO_3_	143	1.3	100-55			22
LT-Cu	Sol-gel, 1	HNO_3_	139	5.0	100-55			22
Cu/CZ1	Sol-gel		253	-	52	47		23
Cu/CZ2	Sol-gel		268	17.8	96	4		23
Cu/CZ3	Sol-gel		241	28.5	97	3		23
Cu/CZ4	Sol-gel		234	31.3	97	3		23
Cu/CZ5	Sol-gel		225	41.2	96	4		23
Cu/ZnO	Sol-gel	Acetates	150	-	64	36	0	20
Cu/Zn 0.1	Ox-co-prec.	Nitrates	39	3.4	36/40			24
Cu/Zn 0.2	Ox-co-prec.	Nitrates	36	14.9	37/46			24
Cu/Zn 0.3	Ox-co-prec.	Nitrates	70	12.6	38/42			24
Cu/Zn 0.4	Ox-co-prec.	Nitrates	45	9.6	37/43			24
Cu/Zn 0.5	Carb-co-prec.				33/38			24
Cu/V	Prec.	Nitrates	185	-	13	67	20	19
Cu/Ag	Co-prec.	Nitrates	281	4.1	81	19	0	25
Ag	Co-prec.	Nitrates	112	-	100	0	0	25
Ag	Impreg.	Nitrates	125	-	70	30	0	26
HT-Ag	Sol-gel, 2	Acetate	99	-	100-55			22
HT-Ag	Sol-gel, 1	Acetate	77	-	100-55			22
HT-Ag	Sol-gel, 1	HNO_3_	125	-	100-55			22
LT-Ag	Sol-gel, 1	HNO_3_	112	-	90-48			22
Au	Co-prec.	HAuCl_4_/ZrO(NO_3_)_2_	185	-	24	76	0	26,27
Au	alloy	Au_25_Zr_75_	47	-	20	74	6	27
Pt	Impreg.	HPtCl_6_/chloride	-	-	5	2	93	28
Pd	alloy	Pd_33_Zr_67_	17	5.6	30	27	43	29
Ni	alloy	Ni_64_Zr_36_	8	-	0	0	100	30
Rh	Impreg.	Nitrate/chloride	-	-	5	32	63	31
Rh	Impreg.	Nitrate/chloride	-	-	0	0	100	32,33
Ru	Impreg.	Ru_(III)_AcAc	-	-	0	0	100	34
Re	Impreg.	-	-	4.1	11	58	29	35
Rh-Mo	Impreg.	Molybdate/chloride	54		0		100	36
Rh-Mo-Li	Impreg.	Molybdate/chloride/nitrate	47		0		100	36
Rh-Mo-K	Impreg.	Molybdate/chloride/nitrate	51		0		100	36
Rh-Mo-Re	Impreg.	Molybdate/chloride/perrhenate	52		0		100	36
Rh-Mo-Co	Impreg.	Molybdate/chloride/nitrate	53		0		100	36
Rh-Mo-Ce	Impreg.	Molybdate/chloride/nitrate	57		0		100	36

^a^ Alloy: controlled oxidation of amorphous alloys, co-prec.: co-precipitation, impreg.: impregnation, ho-prec.; homogeneous precipitation using urea, sol-gel, 1/2: one/two stage sol-gel methodology, ox-co-prec.: oxalate co-precipitation, carb-co-prec.: carbonate co-precipitation. ^b^ Note: product selectivities were obtain under different experimental conditions.

**Table 4:** Recent investigated catalysts for one-pot CO_2_-to-DME process. PM: physical mixing; WM: wet mixing; CP: co-precipitation; IM: impregnation; GHSV: Gas Hourly Space Velocity; P: reaction pressure; T: reaction temperature; X_CO_2__: conversion of CO_2_; Y_i_: carbon-basis yield of *i*-product.

Catalyst	Preparation Method	H_2_/CO_2_	GHSV (NmL∙g^−1^∙h^−1^)	P;T (MPa; °C)	X_CO_2__ (%)	Y_CO_ (%)	Y_MeOH_ (%)	Y_DME_ (%)	Ref.
Cu/Zn/Al	PM	3	3000	5;260	31	2	9.3	19	[145]
HZSM5									
Cu/Zn/Al									
γ-Al_2_O_3_					20	11.6	8	0.4	
Cu/Zn/Al/Zr ZSM5	WM	3	3100	3;260	24.1	7	10.6	6.4	[146]
Cu/Zn/Zr Ga-Sil1	CP	3	1200	3;250	19.0	6.4	4	8.6	[147]
Cu/Ti/Zr ZSM5	WM	3	1500	3;250	15.6	6.1	2.0	7.4	[148]
Cu/Zn/Zr/V ZSM5	CP	3	1500	3;270	32.5	9.1	4.3	19.1	[77]
Cu/Zn/Al/Zr ZSM5	PM	3	6000	5;270	27.5	-	5.0	16	[149]
Cu/Zn/Al/La ZSM5	PM	3	3000	3;250	43.8	0.11	1.9	31.2	[150]
Cu/Mo ZSM5	IM	2	1500	3;240	12.4	2	0.7	9.5	[151]
Cu/Zn/Zr/Pd ZSM5	CP	3	1800	3;200	18.7	2.4	2.5	13.8	[152]
Cu/Zn/Al ZSM5+CNTs	PM	3	1800	3;260	46.2	8.9	16.4	21	[153]
Cu/Zn/Zr FER	CP	3	8800	5;260	23.6	9.2	3.5	10.6	[154]
Cu/Zn/Al	CP	3	750	4;275	35			23	[155]
ZSM5									
Cu/Zn/Al									
γ-Al_2_O_3_					40	-	-	10	
Cu/Zn/Al Amorphous silica-alumina	CP	3	1800	3;270	47.1	12.3	14.7	20.1	[156]
Cu/Fe/Zr ZSM5	PM	5	1500	3;260	28.4	2.2	4.2	18.3	[157]
